# Experimental study on damage of slope under drying and wetting cycle based on DC electric method

**DOI:** 10.1371/journal.pone.0297276

**Published:** 2024-02-23

**Authors:** Ensheng Kang, Zexi Zhao, Haidong Meng, Zihao Zhao

**Affiliations:** 1 School of mining and coal, Inner Mongolia University of Science and Technology, Baotou, Inner Mongolia, 014010, China; 2 College of Resources and Civil Engineering,Northeastern University, Shengyang, 110819,China; Instituto Tecnologico de Aeronautica, BRAZIL

## Abstract

In order to investigate the seepage law and crack development characteristics of dump slopes, as well as the impact on slope stability during drying and wetting cycles, a simulation test slope system was constructed in a rainfall environment, specifically designed to mimic the engineering conditions of dump slope. The apparent resistivity response formula for the seepage and crack development processes was derived based on the three-phase medium theory of rock-soil bodies and Maxwell’s conductivity formula. The geoelectric field characteristics pertaining to slope damage and the corresponding patterns of alteration were comprehensively investigated. The results demonstrate a negative correlation between resistivity and slope water content, with resistivity increasing as cracks develop and decreasing with water infiltration. The progression of crack formation in a rainfall environment on a dump slope can be categorized into three stages: The initial phase involves the saturation of the slope as water content increases. Subsequently, the second phase entails the initiation and expansion of capillary zones, along with the formation of dominant waterways. Lastly, the third phase encompasses the formation and expansion of cracks within the dumping site. The occurrence of sudden changes and abnormal fluctuations in apparent resistivity within a saturated slope signifies the presence of cracks and weak surfaces, leading to gradual and irreversible damage. This phenomenon serves as an indicator of slope damage and can be utilized for the early prediction of slope instability.

## Introduction

The dump slope have loose structure, good permeability and poor anti sliding ability. Under the influence of external factors such as rainfall and vibration, it is very prone to occur slope geological disasters [1]. Research show that cracks and weak plane formed in the process of rainfall seepage is the key factor inducing slope instability, most landslides occur during or shortly after rainfall [2–5]. With the process of rainfall and seepage, cracks in slope form in the cycle of water absorption expansion and water loss contraction, which destroy the integrity of the original structure. At the same time, it also provide good channel for water infiltration, result in the formation of saturated ponding area in slope [6,7]. Water cause the increase of slope weight and the decrease of slope strength, and may lead to the instability and failure of slope [8–10].

Dump slope is a typical man-made granular slope, with large dispersion of particle size and uneven spatial distribution. In the formation process of slope, internal structure of rock-soil body has been reconstructed. The mechanical properties are complex and difficult to obtain. Scholars have carried out many research on rainfall slopes [11–13]. Cho Y et al [14] install electrical sensors and rain gauges on the top of the waste dump slope for monitoring. Study slope deformation based on monitoring results. The study indicate that the top of the waste dump slope undergoes deformation with the weight of the slope increases due to the infiltration of rainwater into the ground. The deformation gradually increases over time and then converges. Yeh, H F et al [15] established a numerical model for rainfall slopes and studied the relationship between rainfall models and slope stability. The results showed that the seepage discharge and the pressure of pore water increase with increasing rainfall, while slope stability increase with decreasing rainfall. Shirshendu L et al [16] studied the stability of coal mine waste dump. A 3D model of the coal mine waste dump was created from UAV imagery and laboratory geotechnical data. The impact of various safety factors on slope stability under earthquake and rainfall conditions was analyzed. Studied the deformation pattern and magnitude of the slope failure. Wang et al [17] improved the conventional solutions of F.S, developed mechanical model for unsaturated inner dump slopes.The study verified the positive proportional relationship between the wetting front and rainfall time. Derived the change process of the transition layer F.S. The model can be used to evaluate the potential sliding surface under rainfall conditions. There is a certain lag in the time between internal damage and whole failure of slope under the influence of external actors such as rainfall. Existing monitoring methods such as borehole exploration and displacement monitoring are difficult to obtain structural change in the slope timely. Unable to analyze the internal damage of slope under drying and wetting cycle quickly, non-destructively and carry out early warning of slope disasters effectively.

With the development of geophysical prospecting techniques, study the medium conditions, spatial distribution law and action characteristic mechanism of geophysical field under the influence of drying and wetting cycle in dump slope, and establish the identification method of geophysical field of seepage and crack development can effectively improve the prediction accuracy and efficiency [18–20]. Liu et al. [21] established soil resistivity test methods under conventional and high gravity environment based on high-density electrical method, which verified the effectiveness of high-density electrical method in detecting soil moisture migration. Du et al. [22] detected the soil resistivity with the help of high-density electrical method, established the mathematical relationship model between resistivity and moisture content, and verified the practicability of the model in describing soil moisture content. Ou et al. [23] used resistivity imaging technology to test the spatio-temporal evolution of surface soil fracture caused by underground mining. Discovered that dynamic fracture always develop ahead of the current working location. Proved the sensitiveness of electrical resistivity imaging technology to the change of soil micro structure. Ismail et al. [24] determined the subsurface resistivity distribution of the study area and map the risk potential zone of landslide in the future. Discover that weak zones are characterized by the large proportion of high water saturation zones and low resistivity values.

Therefore, dump slope under rainfall is taken as a whole to study the resistivity change laws and characteristics of slope seepage and crack development during rainfall seepage, study the resistivity signs of slope damage, find out the corresponding relationship between the internal damage of the slope and the overall failure can realize the supplement to the slope disaster mechanism and prediction methods of dump slope under rainfall condition [25–27].

In this paper, a dump slope test platform under the condition of rainfall was made according to the similarity theory. Detected the resistivity change by Direct Current (DC) electric method. Trends and characteristics of slope resistivity variation during rainfall were analyzed. Mechanism of changes in resistivity under drying and wetting cycle were deduced. The development process and resistivity evolution law of seepage and crack were compared by conventional inversion and time-lapse inversion. The findings can provide scientific theory and technical support for disaster prevention, reduction and safety management.

## Experimental methods

### Assumptions for model establishment

The research object is the characteristics and law of seepage evolution and crack development in the dump slope during rainfall. Based on previous studies, the test model was simplified as follows to build the physical model of slope [28,29].

1. A single slope step was selected as the reference for the construction of similarity model, and the sandy soil with different particle sizes and different content ratio were selected to simulate slope.
2. In order to reduce the impact of rainfall on slope surface erosion, low intensity rainfall was selected, and the influence of air pressure of rock-soil body on rainfall seepage was ignored in this study.
3. The influence of physical model boundary effect on apparent resistivity measurement was ignored.

### Experimental steps

In this study, the macro dimensions of the model slope and the relevant parameters of the test materials were determined according to the similarity theory in combination with the actual production situation. The test materials were screened and classified according to the similarity ratio, density and permeability. The slope was stacked and compacted layer by layer according to the ratio, and the natural repose angle was selected as the slope angle. We conducted multiple tests before the formal test combinate with the engineering background and previous studies. It is found that the shoulder and front edge of the top of the slope are greatly affected by the free surface under the influence of rainfall seepage where cracks likely occur. Therefore, the resistivity measuring line were selected to be arranged along the horizontal direction at the top of the slope.

**Fig 1:**
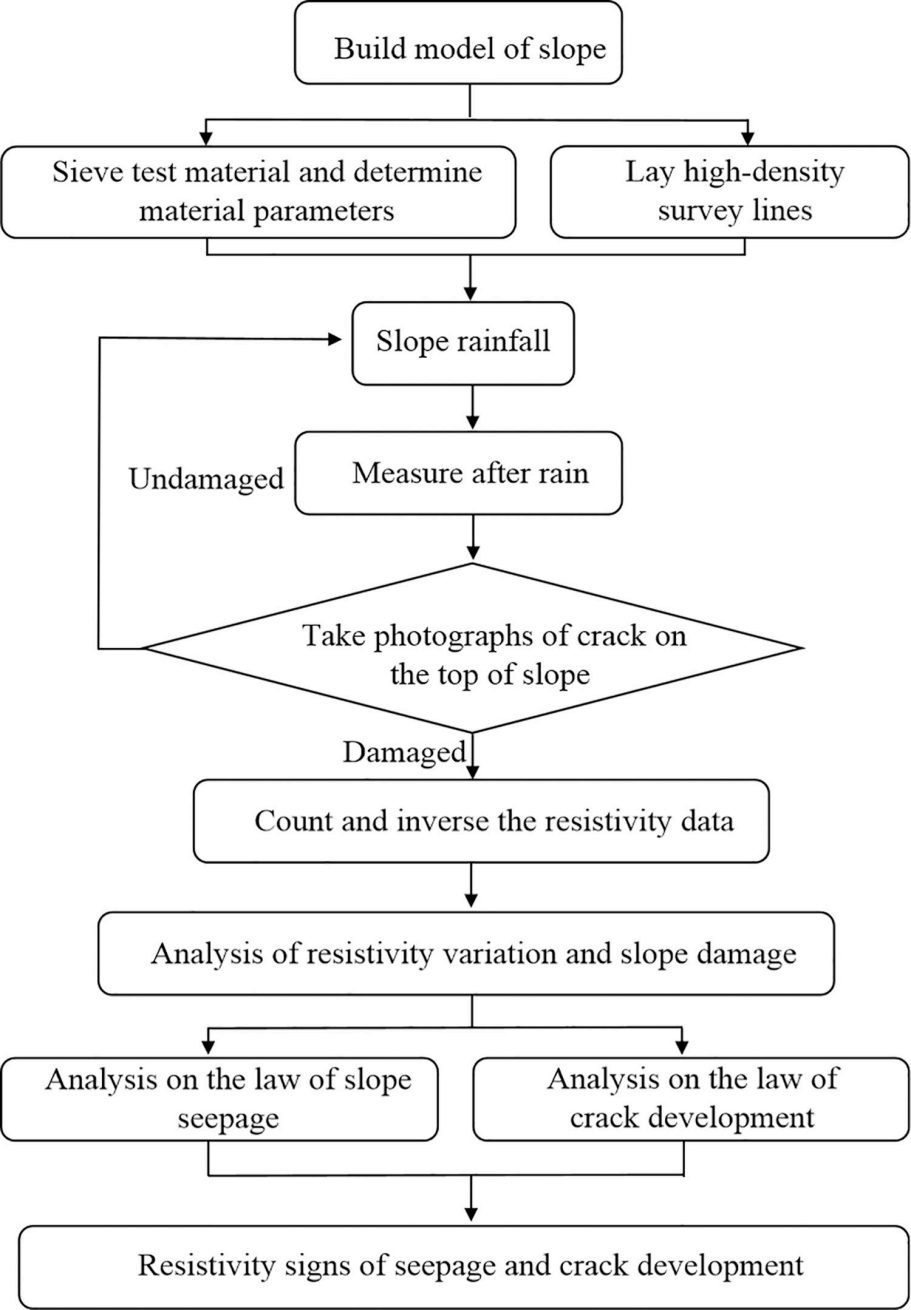
Test and research scheme.

The experiment was carried out in a cycle mode of concentrated rainfall and measurement after stop rainfall. Each rainfall time is 10 minutes, and the measurement time for no rain is 3 minutes. The mobile rainfall device and control of rainfall speed can ensure uniform water flow at different positions on the slope top, the water can fully penetrate into the slope. Repeat rainfall and measurement process until landslide occurs. By observing change of apparent resistivity inside the slope, and comparing with the external damage of the slope, the characteristics and change laws of rainfall seepage and crack development process were analyzed. The relationship between resistivity change and damage of slope under drying and wetting cycle were studied. The research scheme is shown in (Fig 1).

### Experimental model and materials

The project background slope is located in Bayan Obo, Baotou City, Inner Mongolia. Located in a hilly area, the terrain is gentle and open. Hydrogeological conditions of the site are simple with good runoff discharge conditions, and recharge source of groundwater is mainly atmospheric precipitation. The waste dump is mainly composed of silty clay, mixed clay, coarse sand, and gravel layer. Coarse grain layer increases from the top to the bottom. Slope angle is the natural resting angle. Collapse failure occurred at the bottom of slope in some areas.

In the test, the reduced scale of dimension C_L_ was selected as 30, the reduced scale of density C_γ_ was selected as 1.5. According to the similarity criterion, reduced scale of the parameters adopted in the model test are shown in Table 1, with subscript i is parameters of in-situ slope and subscript m is parameters of model slope.

**Table 1:** Reduced scale of the parameters adopted in the model test.

Parameters	Definition	Ideal reduced scale
Dimension	C_L_ = L_i_/L_m_	30
Density	C_γ_ = γ_i_/γ_m_	1.5
Friction coefficient	C_φ_ = φ_i_/φ_m_	1
Poisson’s ratio	C_μ_ = μ_i_/μ_m_	1
Elasticity modulus	C_E_ = E_i_/E_m_	45
Cohesion	C_C_ = C_i_/C_m_	45

**Fig 2:**
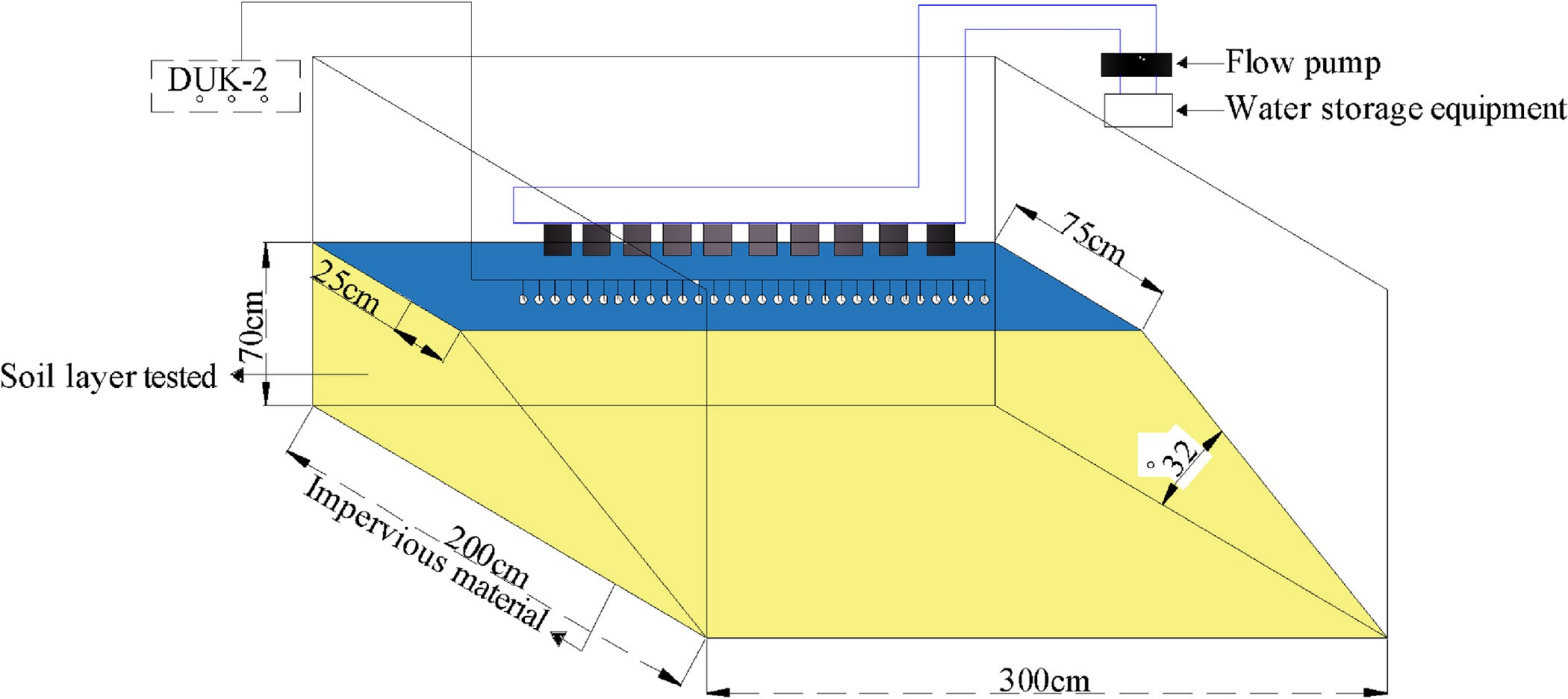
Design diagram of test model.

Based on similarity ratio, length of the test model waste dump is 300cm, width of the platform is 200cm, width of top is 75cm, height of the slope is 70cm, angle of slope 32° is the average value angle of repose. The test model design is shown in (Fig 2).

In order to facilitate the layout of survey lines and on-site monitoring, the model adopted open side structure. In order to reduce the interference to the measuring equipment, insulating materials were set at the bottom of the model. At first small stones, river sand and screened soil were dumped and compacted layer by layer according to the mass ratio of 1:3:5, and laid to a height of 60cm, then laid 10cm screened soil to a design height of 70cm. The performance of model material parameters is shown in Table 2.

**Table 2:** Parameters of test materials.

	Soil	Fluvial sand	Small grain stone	Mixture
Dry density (g·cm^-3^)	1.23	1.34	1.35	1.30
Wet density (g·cm^-3^)	1.66	1.73	1.95	1.84
Permeability coefficient (cm·s)				4.06×10^−5^

Build process of test model simulated the construction of dump slope. The original mechanical structure of rock-soil body changed during the formation of dump slope. In general, the horizontal layers of the slope are approximately evenly distributed, forming certain sorting along the vertical direction, the internal structure is clear. The upper and middle parts of the slope are mainly fine particles, and the lower parts are mainly coarse particles. The adhesion between particles in the slope increases with the increase of fine particle content, the friction force decreases with the increase of fine particle content. Before rainfall, the water content of the slope is low, there are no faults, cracks, aquifers, weak plane, et al in the slope, the overall resistivity is large. Some low resistance area of the shallow slope is caused by the watering to strengthen the contact between the electrode and the soil. Under the influence of layered compaction and slope dead-weight, the porosity of the bottom layer of the slope is lower than that of the middle layer, appeared as the resistivity of the middle slope is higher than bottom slope. Due to the uneven effect of compaction, an obvious "defect" was formed in the middle position of the survey line, and the resistivity was relatively high.

### Experimental equipment

The test system consists of slope model, water supply device, movable artificial rainfall equipment, measuring equipment for water content, photographic equipment and high-density electrical method detection system. The detection system adopted DUK-2a high-density electrical method measurement system of Chongqing Geological Instrument Factory, including Dzd-6a multi-functional DC electric method instrument, Mis-120 multi-channe electrode converter and modified monitorin electrode. In order to reduce runoff erosion, mobile rainfall equipment with drip arrow nozzles was selected. Rainfall intensity was set as 0.21mm/min.

### Fundamental

#### DC resistivity theory

High-density electrical method is a widely used DC electric method with large amount of information collected and high data accuracy, which is suitable for detecting the internal structure of non-uniform objects. Based on the electrical property difference of rock-soil body. Emit DC electrical signals to the slope underground through electrode *A* and electrode *B*, measure the potential difference between electrode *M* and electrode *N*, calculate the resistivity value of the underground target measurement point according to Eq (1) [30].


$$\rho =\frac{RS}{L}$$ (1)

Where *ρ* is the resistivity, *R* is the resistance of the material, *S* is the cross-sectional area of the material, and *L* is the length of the material.

In electrical exploration, apparent resistivity *ρ*_*S*_ is often used to describe the change in electrical conductivity of rock-soil body, as shown in Eqs (2) and (3).


$$\rho_{S}=K∙\frac{\Delta U_{MN}}{I}$$ (2)

$$\Delta U_{MN}=\int_{N}^{M}E_{MN}∙dl=\int_{N}^{M}j_{MN}∙\rho_{MN}dl$$ (3)

Where *K* is the coefficient of the electrical prospecting device, Δ*U*_*MN*_ is the potential difference between the measuring electrodes *M* and *N*, and *I* is the current. *E*_*MN*_ is the electric field strength between the measuring electrodes, *j*_*MN*_ is the current density between the measuring electrodes *M* and *N*, *ρ*_*MN*_ is the resistivity between the measuring electrodes, and *dl* is the length unit between the measuring electrodes *M* and *N*.

**Fig 3:**
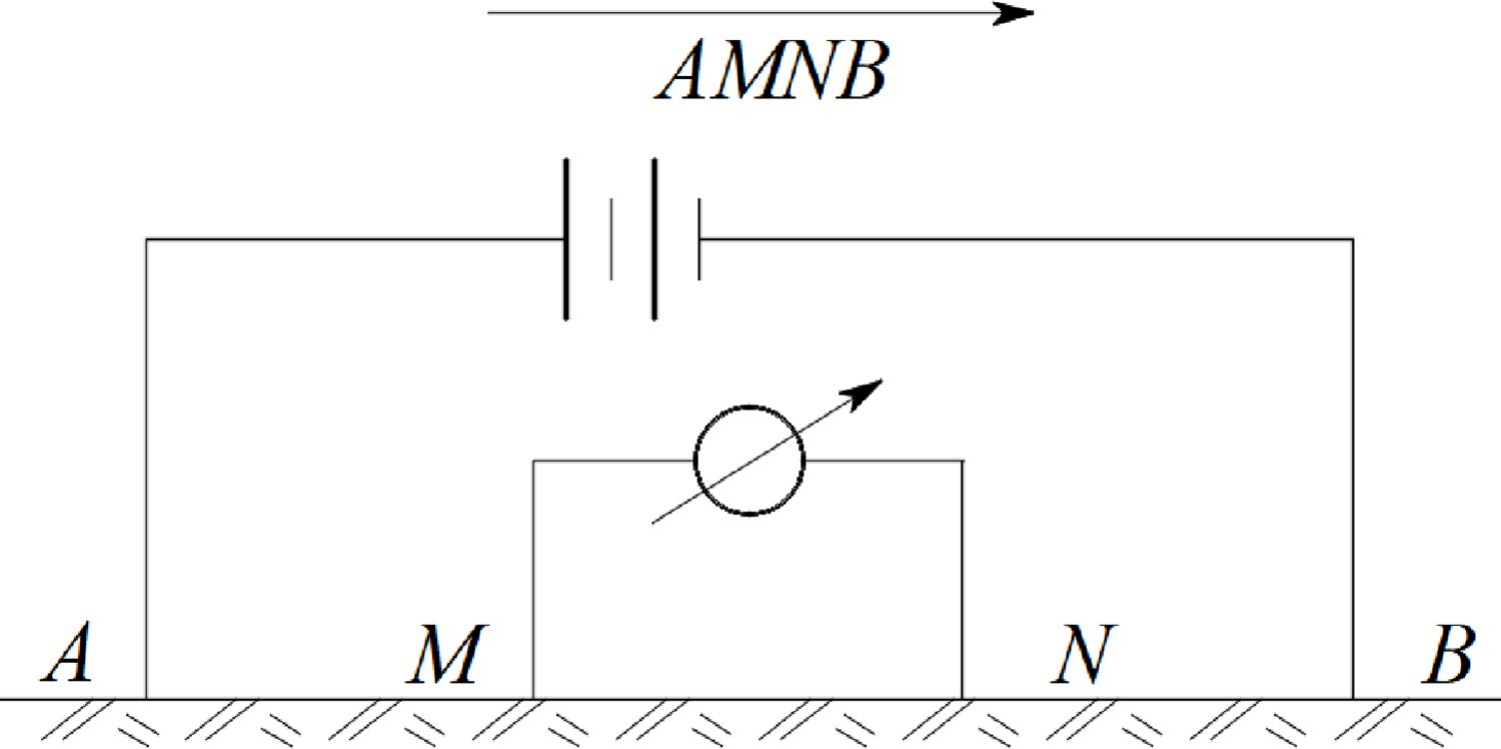
Wenner measurement method.


The Wenner method used in this test is set symmetrically with four poles, *O* is the center point, *M*、*N* and *A*、*B* are symmetrical on both sides of *O*, *AM* = *MN* = *NB*, which *ρ*_*S*_ can be expressed as Eqs (4–6). The measurement method is shown in (Fig 3).

$$\rho_{S}=K∙\frac{\Delta U_{MN}}{I}=\frac{2\pi }{\frac{1}{AM}-\frac{1}{BM}-\frac{1}{AN}+\frac{1}{BN}}∙\frac{\Delta U_{MN}}{I}$$ (4)

$${\Delta U_{MN}=U}_{M}-U_{N}=\frac{I\rho }{2\pi }(\frac{1}{AM}-\frac{1}{BM}-\frac{1}{AN}+\frac{1}{BN})$$ (5)

$$K=\frac{2\pi }{\frac{1}{AM}-\frac{1}{BM}-\frac{1}{AN}+\frac{1}{BN}}$$ (6)

### Time-lapse resistivity theory

Time-lapse resistivity inversion adds a time constraint term on the basis of conventional resistivity inversion algorithm, focus on the change of detection data at different times and the difference of electrical parameters of underground media. Takes the inversion results as the initial data of next inversion, and improves the inversion iteration speed and imaging accuracy by introducing regularization functions in space and time domains, as shown in Eq (7) (Sun et al. 2019)[31].


$$\left(J_{i}^{T}R_{d}J_{i}+\lambda_{i}W^{T}R_{m}W)\Delta r_{i}^{k}=J_{i}^{T}R_{d}g_{i}-\lambda_{i}W^{T}R_{m}Wr_{i-1}^{k}-\beta_{i}\lambda_{i}W^{T}R_{i}V(r_{i-1}^{k}-r_{i-1}^{0}\right)$$ (7)

Where, *J* is the Jacobian partial derivative matrix, *i* is the number of iterations, *g*_*i*_ is the logarithmic difference matrix between the measured apparent resistivity and the calculated apparent resistivity, *W* is the smoothing filter parameter matrix, *λ* is the damping coefficient, *R*_*d*_ and *R*_*m*_ is the weight matrix, $r_{i-1}^{0}$ and $r_{i-1}^{k}$ is the initial value of the model and the k-th time value, $\Delta r_{i}^{k}$ is the model residual of the ith iteration, *V* is the weight matrix between models at different times, *β* Is the weight coefficient [32].

According to the distribution and evolution characteristics of the medium apparent resistivity inside the slope, calculate the residual value *Δρ* of the resistivity of the inversion data at different times at the same location under the survey line, describe the change characteristics of resistivity with time, as shown in Eq (8) [33].

$$\Delta \rho =\rho_{i}-\rho_{0}$$ (8)

where *ρ*_*i*_ and *ρ*_0_ are the resistivity value at time *i* and reference time respectively

### Archie formula

According to the extended Archie empirical formula, the correlation between the resistivity, porosity, and water saturation of water bearing rock-soil body can be expressed as Eq (9) [34].


$$\rho =a\rho_{w}\emptyset^{-\alpha }S^{-\beta }$$ (9)

Where *ρ* is the formation resistivity of rocks; *α*、*β*, and *α* are constants, ∅ is porosity and *S* is the volume fraction of pores filled with water, *ρ*_*w*_ is the resistivity of the pore fluid.

### Experimental process and result analysis

#### Slope damage process under drying and wetting cycle

**Fig 4:**
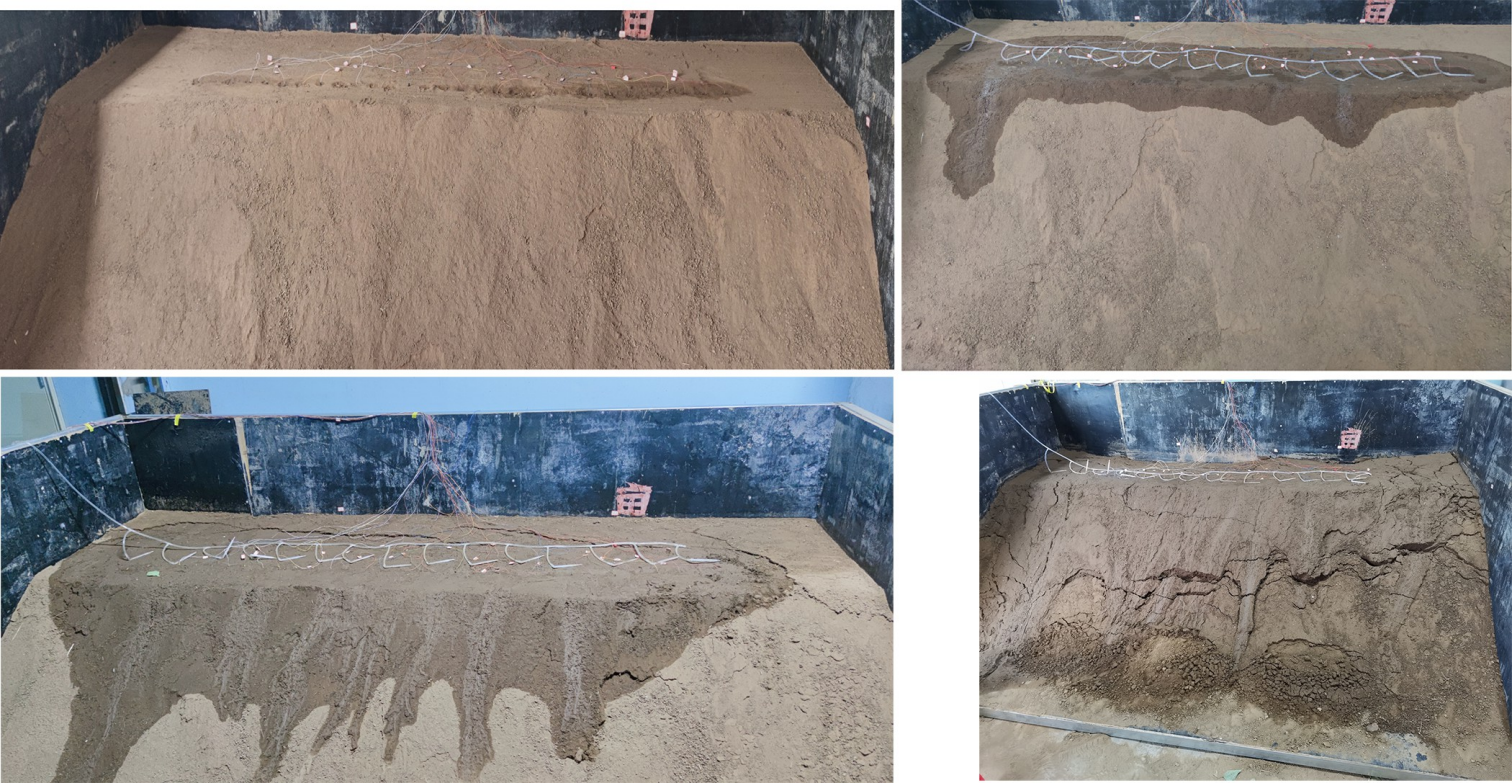
Rainfall process of model slope.

According to the observation of slope deformation and failure during rainfall and measurement cycles, a total of 111 rainfall events were conducted in the experiment. (Fig 4) show the change process of slope at equidistant time during rainfall. With the increase of rainfall and measurement period, the slope is gradually wetted, caused cracks. Structure of the slope were damaged under drying and wetting cycle until slope failure.

Before rainfall, the model material was uniform and the slope soil was relatively dry. After the 35th rainfall, the infiltration had significant difference. Water at the top of the slope gradually diffused to the shoulder, and the infiltration rate at the left side was faster than that in other areas. After the 70th rainfall, large amount of runoff erosion occurred on the slope surface, and obvious tension cracks appeared at the rear of the slope top. Water flowed into slope through cracks on the slope top. After the 105th rainfall, due to the increase of slope weight, the transverse cracks on the top of the slope were further widened and gradually developed to the shoulder of the slope, and the network secondary cracks on the top of the slope were developed. A number of crisscross cracks were formed on the slope, obvious traction slip was formed in the middle of the slope, and collapse appeared at the toe of slope.

The rainfall seepage slope conforms to the saturated-unsaturated seepage process. During the measurement after stopping rainfall, the loss of water in slope cause uneven shrinkage of in the rock-soil body. When the shrinkage stress is greater than the tensile strength, shrinkage cracks were generated, and the slope was decomposed into different pieces. In the process of rainfall, water seeped into the slope along cracks. Increase of water content resulted in the rapid decline of slope strength. Water absorption and expansion of rock-soil body cause loose of particle arrangement and increase of pore volume. Expansion cracks were generate when the expansion stress is greater than the tensile strength of rock-soil. With the cycle of rainfall and drying, cohesion and friction angle of rock-soil body were continuously reduced, and the slope strength is further reduced. With the increase of infiltration, the saturated areas in the slope develop and connect. Water soften the soil, weak plane form in slope. Stability of slope decreased and finally forms landslide under multiple actions such as increase of dead-weight, change of pore water pressure and weakening of slope toe support [35].

### Changes of resistivity inside the slope during damage process

**Fig 5:**
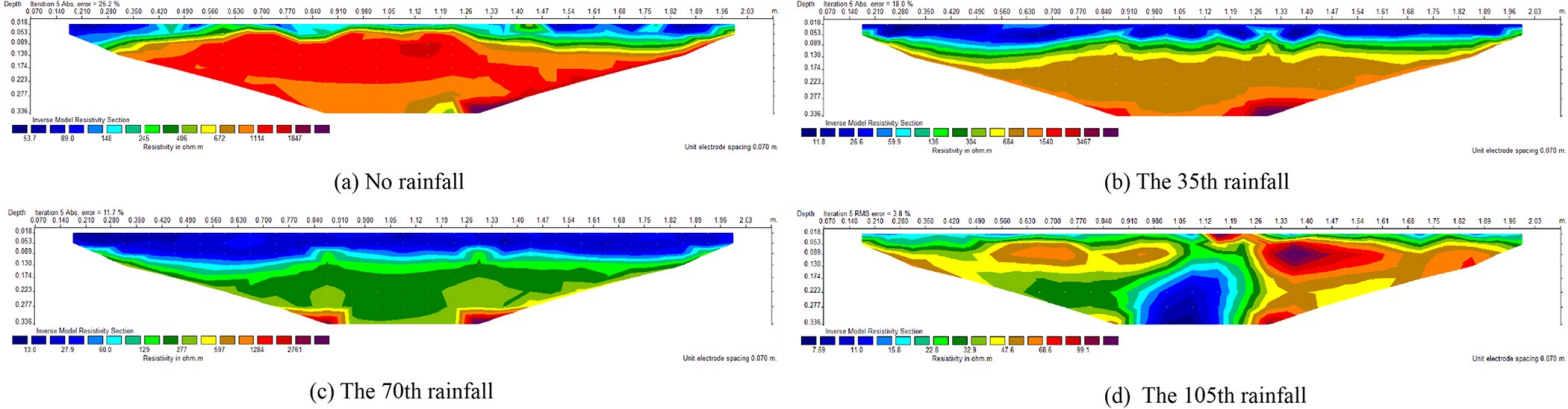
Resistivity inversion of line area.

Fig 5 is the inversion diagram of resistivity changes under survey line at the corresponding rainfall time of (Fig 4). It can be seen that there were significant changes of the resistivity inside the slope during failure process. Before rainfall, the overall resistivity showed high resistance. As rainfall increased, resistivity gradually decreased from shallow to deep layers. In later stage of rainfall, shallow slope exhibit relatively high resistance.

**Fig 6:**
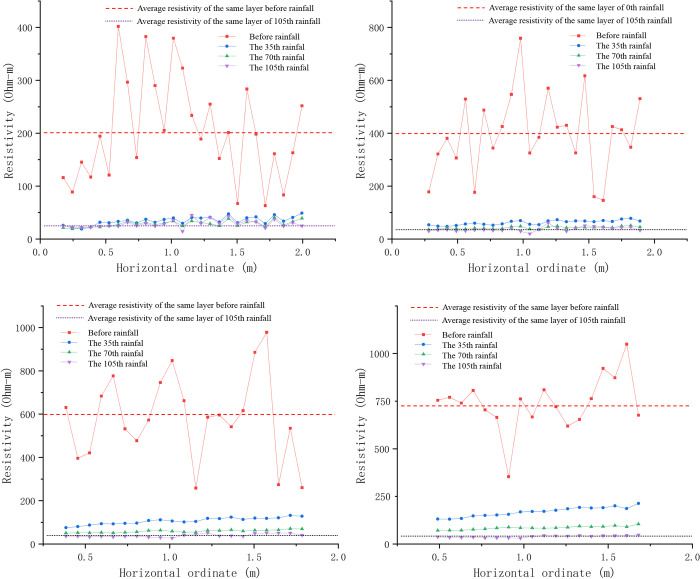
Resistivity of rainfall process at typical depth.

The resistivity of water is fixed value under test conditions according to Archie empirical formula (Eq 9). The resistivity of water bearing slope is related to porosity and saturation. With the increase of rainfall, water content and saturation of the slope increase, which appears as the overall apparent resistivity of the slope decrease after rainfall. Affected by evaporation and seepage, rock-soil body gradually develop cracks, resulting in an increase in the porosity (crack) rate inside the slope. Water content and saturation of rock-soil body fluctuate in the drying and wetting cycle. Apparent resistivity of slope change constantly under the coupling of crack development and water infiltration.

(Fig 6) is the resistivity-monitoring points variation curve at typical depths. Average apparent resistivity of each layer increase with depth before rainfall. The reason is that original structure of the rock-soil body have been changed during the formation of slope. The particles in deep layers of slope are larger, so the porosity of deep slope is greater than shallow slope. After the 35th rainfall, average apparent resistivity at depth of 0.035m reached 35 Ω· m. At this time, the slope at this depth is close to saturation, and there were no obvious cracks on the surface of the slope.

There are significant differences in the speed of resistivity change at the same depth and different horizontal coordinates. Resistivity decrease in the left slope is faster than other areas. It can be observed from (Fig 4) that the seepage of left slope extended to the shoulder. The reason is that in the process of accumulation and reconstruction, rock-soil particles in the dump slope are unevenly distributed under the layered compaction. In particular, the compactness of shallow slope is poor, structure and strength of rock-soil body at different locations are obviously different, leading to the change of seepage flow field. The fine particles in the area with dense particle distribution move towards deep slope under the action of dissolution and drag, and the peak of flow velocity is concentrated [36]. Sedimentation occur during the migration of fine particles, block the internal pores of rock-soil body, result in the reduction of local permeability of rock-soil body [37], and then the seepage direction changes.

Based on the difference of resistivity change speed, it can be judged that the left slope has a higher proportion of small particles and good compaction. Porosity of the left slope is relatively small, while porosity of the central area is relatively large. As rainfall process, the difference in resistivity between different locations of slope gradually decreases.

After the 105th rainfall, average apparent resistivity of each layer was close to the saturation resistivity of the test slope. After reaching saturation state, the resistivity of shallow area exhibits significant fluctuations, corresponding to the location of cracks on the slope top. The fluctuation of resistivity values in deep slope is relatively small. There for, it is possible to infer the internal structural damage of slope under the action of drying and wetting cycles based on the changes of resistivity at different locations.

## Discussion

### Resistivity characteristics under drying and wetting cycle

**Fig 7:**
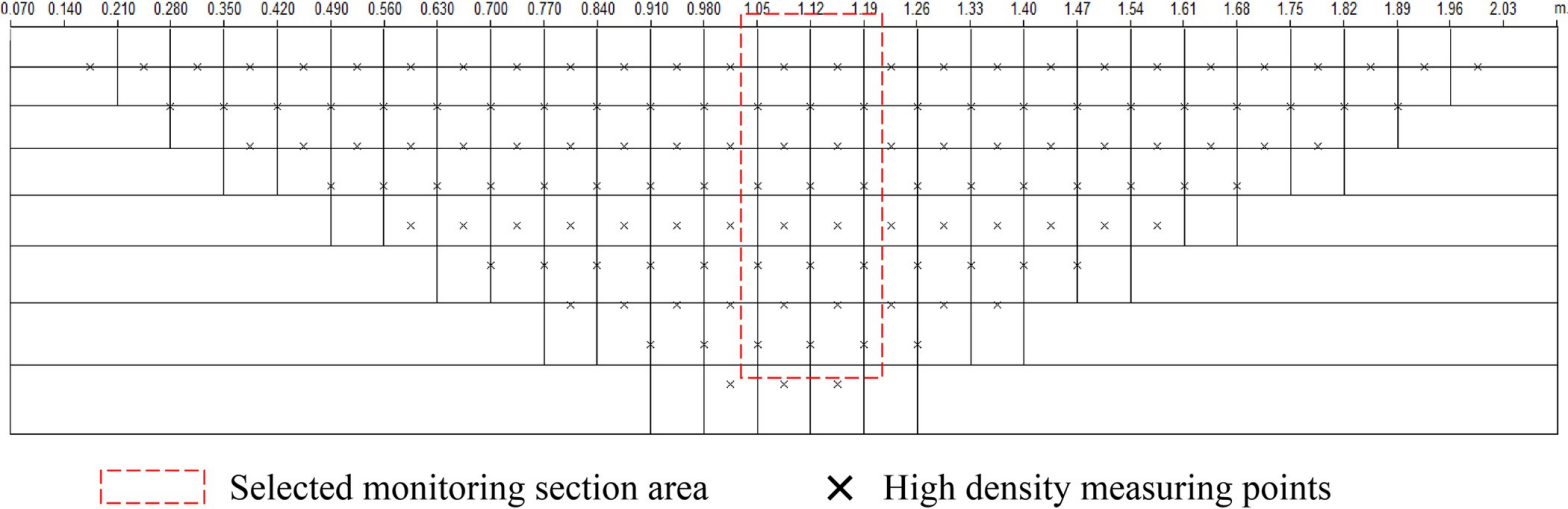
Distribution of detection points.

**Fig 8:**
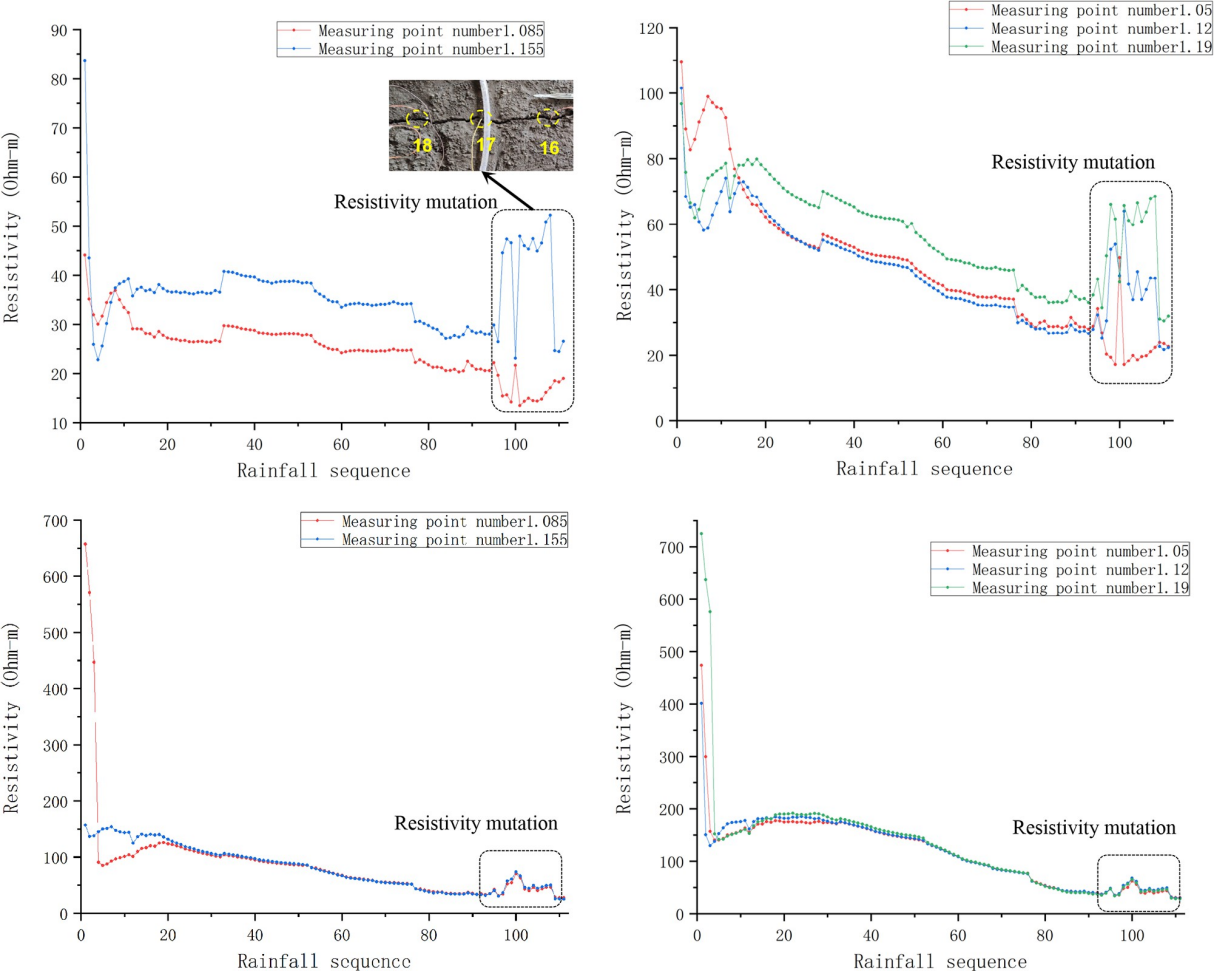
Resistivity characteristics of rock and soil damage.

Based on experimental observations results, taking the range of abscissa 1.05–1.19m (Fig 7) as an exemple, the relationship between resistivity and rainfall period (Fig 8) is drawn to study the resistivity characteristics of water migration and crack development under drying and wetting cycle.

It can be seen from (Fig 8) that resistivity of each layer fluctuated and gradually decreased with change of water content in rainfall process.

The resistivity of slope decreased significantly for the first time after the 5th rainfall. By comparing resistivity changes at different depths, it can be found that the first rapid decline time of resistivity increase with the increase of depth. It is because the pores of the shallow slope rock-soil body were rapidly filled to saturation by water and the conductive ions in the slope enter the pore water, conductivity of upper layer slope enhanced rapidly. Affected by saturation of slope and increase of the dead-weight, lower layer of slope were compacted, connectivity of rock-soil body was enhanced, resistivity decreased rapidly.

It can be seen from Fig 8A and 8B that after the 95th rainfall, the resistivity of the shallow slope had obvious sudden changes and fluctuation. The max amplitude is 40 Ω·m. Obvious cracks appeared at the measuring line on the top of slope after the 100th rainfall. From Fig 8C and 8D, it can be seen that after the 95th rainfall, resistivity of other layers also appeared sudden changes of different amplitudes, the change amplitude decrease with the increase of depth. The results show that the corresponding areas within the slope had been damaged to varying degrees before the formation of cracks on the slope top. In other words, after the 95th rainfall, cracks were formed from the shallow slope and gradually developed to deep and surface of slope. The slope soil shrank and deformed under the action of water evaporation and seepage. When the tensile stress generated by shrinkage is greater than the tensile strength of rock-soil body and the side stress of dead-weight, the slope will produce large shrinkage cracks, and the apparent resistivity will increase dramatically. When rainfall again, the rock-soil body will absorb water and expand. When the expansion stress is greater than the tensile strength and the lateral stress of dead-weight, shallow and short expansion cracks will be generated, and the apparent resistivity value will increase slightly. In the process of drying and wetting cycle, cracks development and water infiltration alternate along the crack, which showed the fluctuation of apparent resistivity in the corresponding area.

**Fig 9:**
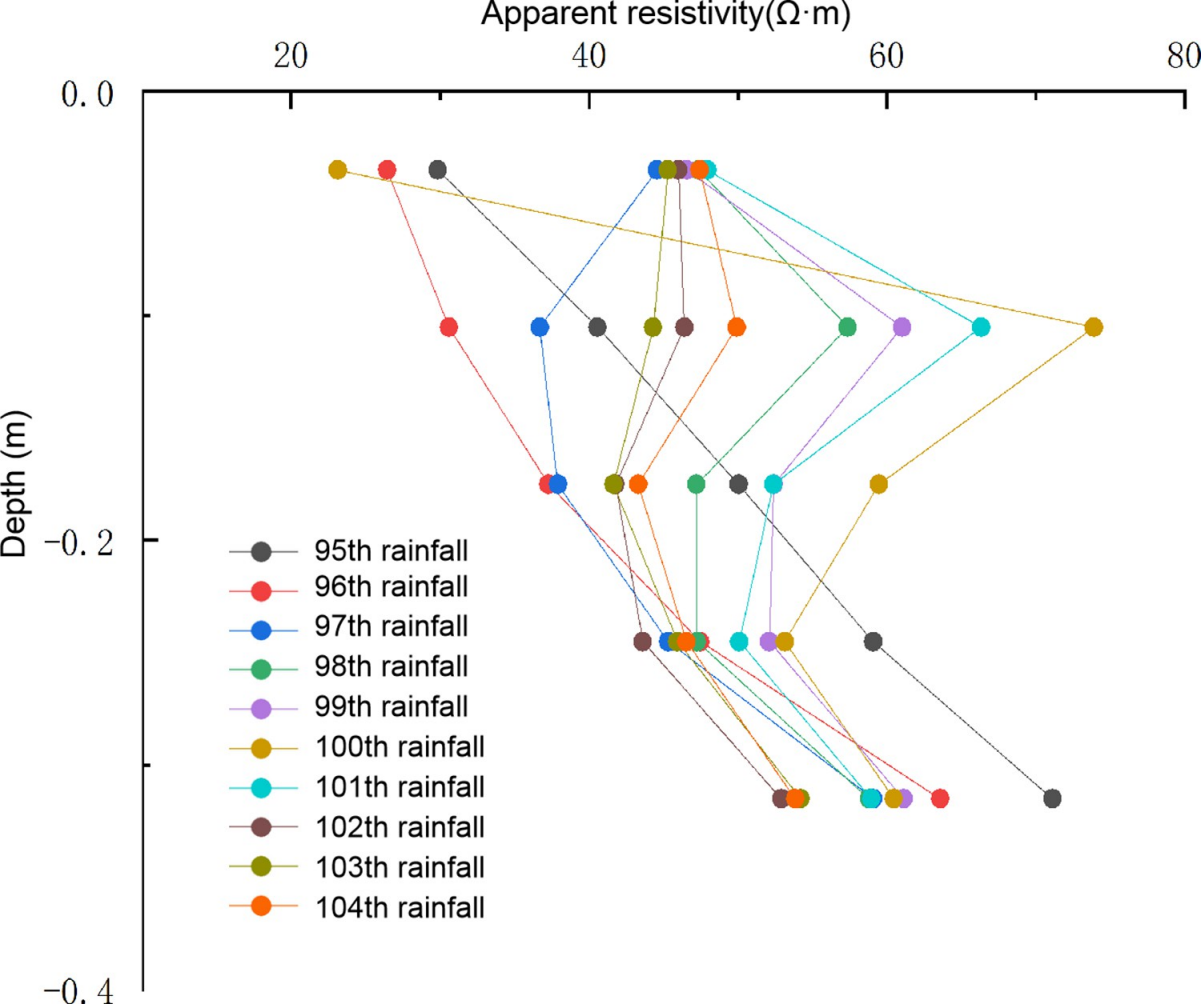
Relationship between apparent resistivity and depth.

It can be seen from (Fig 8) a that the apparent resistivity of the two measuring points (abscissa 1.085m and 1.155m) have same change trend before the 95th rainfall, and have opposite trend after the 95th rainfall. The reason is that the structure of rock-soil body in the two places changed in different forms during the seepage process. Apparent resistivity increases in the process of cracks development. After the formation of cracks, the water forms a dominant inflow seepage, and the apparent resistivity decreases. It can be judged that crack developing at 1.155m and water infiltrating at 1.085m after the 95th rainfall, crack developed from 18th point to 17th measuring point. According to (Fig 8C and 8D), the trend of apparent resistivity of each layer of slope below 0.105m depth is consistent. With the increase of rainfall and slope moisture content, the resistivity of each layer finally decreased to about 40Ω·m, which is close to the saturated moisture content of soil. After the test, the slope was excavated. It is verified that the soil mass of the slope was gradually saturated in the process of rainfall and seepage, and a weak plane formed in the slope.

(Fig 9) shows the relationship of apparent resistivity with depth at the abscissa of 1.155m during the 95th to 104th rainfall cycles. The depths of measuring point correspond to the first, third, fifth, seventh, and ninth layers of resistivity exploration. From the overall trend, the variation amplitude of shallow resistivity is significantly greater than that of deep resistivity. Resistivity of the area below the fifth layer (depth 0.175m) varies slightly with the rainfall cycle, indicated that the critical depth of rainfall seepage in this test is around 0.175m, impact of rainfall seepage and evaporation decreased below this depth.

During the 95th and 96th rainfall, apparent resistivity increased with the increase of depth. After the 97th rainfall, apparent resistivity of the first layer (0.035m depth) increased, while apparent resistivity of the third layer (0.105m depth) decreased. Apparent resistivity decreased first and then increased with increase of depth. Changes in the relationship between resistivity and depth at different rainfall times indicate that water in the monitoring section seeps into the depth slope under gravity and water head, rock-soil body shows low resistivity as the water content increases. Rock-soil body of the slope adjacent to the first, third layers gradually damaged and developed cracks after the 96th rainfall, resulting in an increase in the apparent resistivity of the rock-soil body. After the 98th rainfall, apparent resistivity of each layer showed increased first and then decreased with the increase of depth. After the 100th rainfall, there was a significant mutation in the resistivity above 0.175m, with the apparent resistivity of the first layer dropping to the minimum value of 23Ω·m, and the apparent resistivity of the third layer reaching the maximum value of 74Ω·m. It was observed that crack damage developed to the top of the slope. It indicated that after the formation of damage inside the slope, cracks gradually developed towards the depth and top of the slope, and the apparent resistivity increases. Cracks provide good water channel for seepage, and the apparent resistivity of shallow areas decreased under the coupling effect of cracks and seepage. During drying-wetting cycle after the 101th rainfall, the variation amplitude of resistivity at various depths significantly weakened, indicated that after the formation of cracks, water seeped into the interior of the slope along with cracks, and the rock and soil at each layer of the slope tend to be saturated.

### Relationship between resistivity and water content

**Fig 10:**
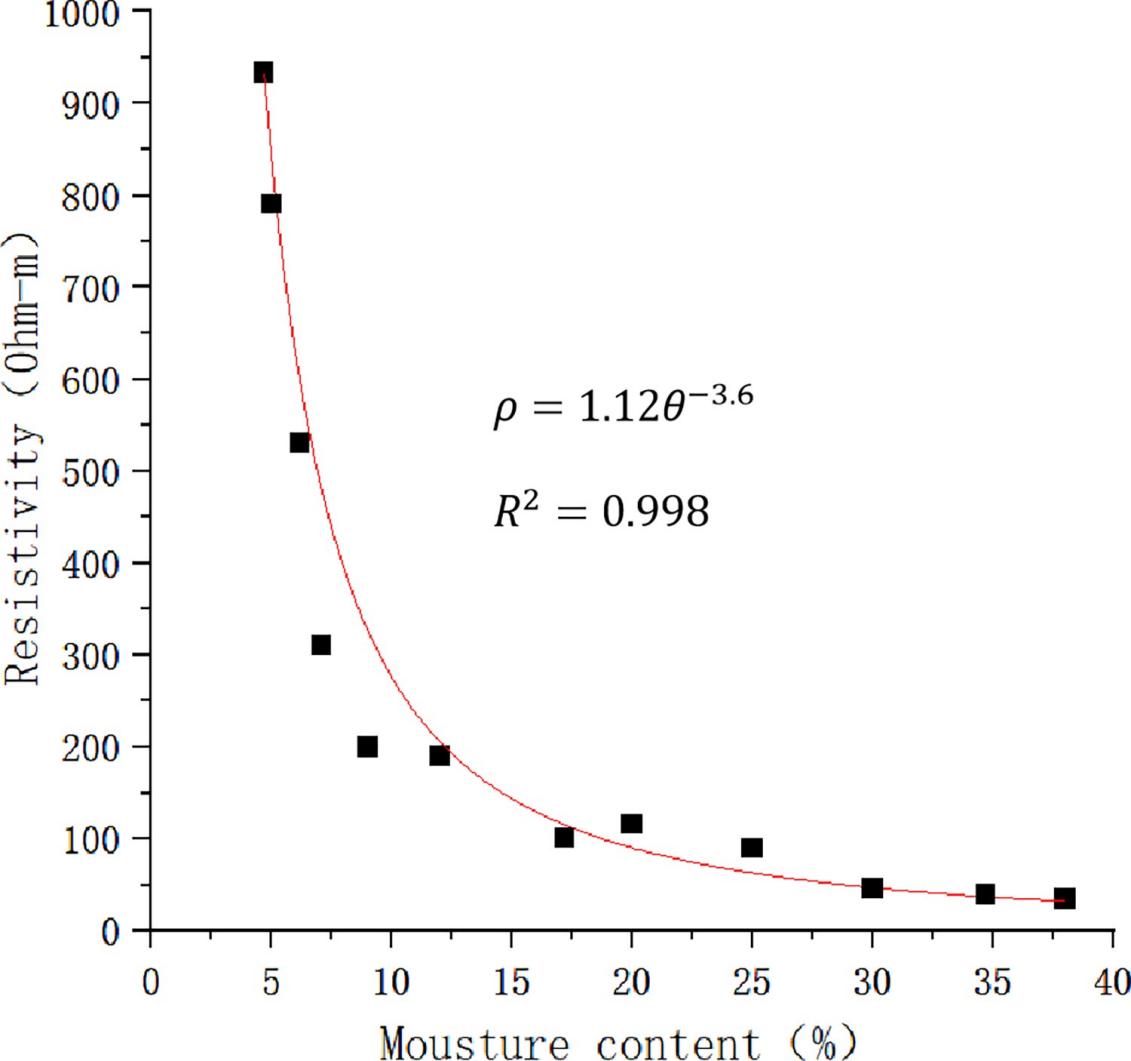
Relationship between slope resistivity and water content.

The resistivity of slope is related to physical properties such as material composition, density, moisture content and pore structure of soil mass, chemical properties of seepage fluid, external ambient temperature and other factors. Among these, water is the mainly influence factor. Research shows that there is an obvious negative correlation between resistivity and water content. Water content and saturation of rainfall slope increase with rainfall seepage. Charge mobility magnify with increase of water content and saturation of rock-soil body, apparent resistivity of slope decrease gradually. Therefore, the change of water content in different stages of the survey line area can be described by the change of apparent resistivity. According to monitoring data, scatter plot (Fig 10) of the relationship between resistivity and water content of slope rock-soil body is drawn, and the Eq (10) is selected to fit the relationship between resistivity and water content [38].


$$\rho =a\theta^{-b}$$ (10)

Where, *ρ* is resistivity; *θ* is the water content; *a* and *b* are constants.

The main existing form of water in soil changes with the increase of water content. Charge mobility increase with the increase of water content, and resistivity decreases with rainfall seepage process. At initial stage of rainfall, resistivity drops rapidly, the form of water in soil changes from adsorbed water to capillary water, and the current changes from electric double-layer conduction along the surface of soil particles to conduction along the surface of soil particles and the series path conduction of water and soil [39]. With the increase of the internal conductive path, the ion mobility increases and the resistivity decreases rapidly. With the increase of water content, the continuity of pore water is enhanced, the current is mainly conducted along the continuous pore water, and the reduction rate of soil resistivity slows down. When the water content is greater than 20%, the main existing form of water is gravity water, the continuity of pore water is good [40], and the soil tends to be saturated. The increase of water content has little effect on the continuity of pore water, the current is mainly conducted by pore water, and the soil resistivity tends to be stable. The test results showed that when it was close to saturation the water content of slope was 34.7% and the corresponding resistivity was 39.4 Ω∙M.

### Resistivity variation mechanism of slope

As shown in Figs 8 and 10, the appearance resistivity can describe the changes of underground media. Archie formula (Eq 9) provide a theoretical basis for the variation of resistivity in water-bearing rock masses. However, Archie formula and related research is based on water-bearing sandstone, which have shortcomings in explaining changes of resistivity in the slope caused by newly formed cracks and the seepage process.

**Fig 11:**
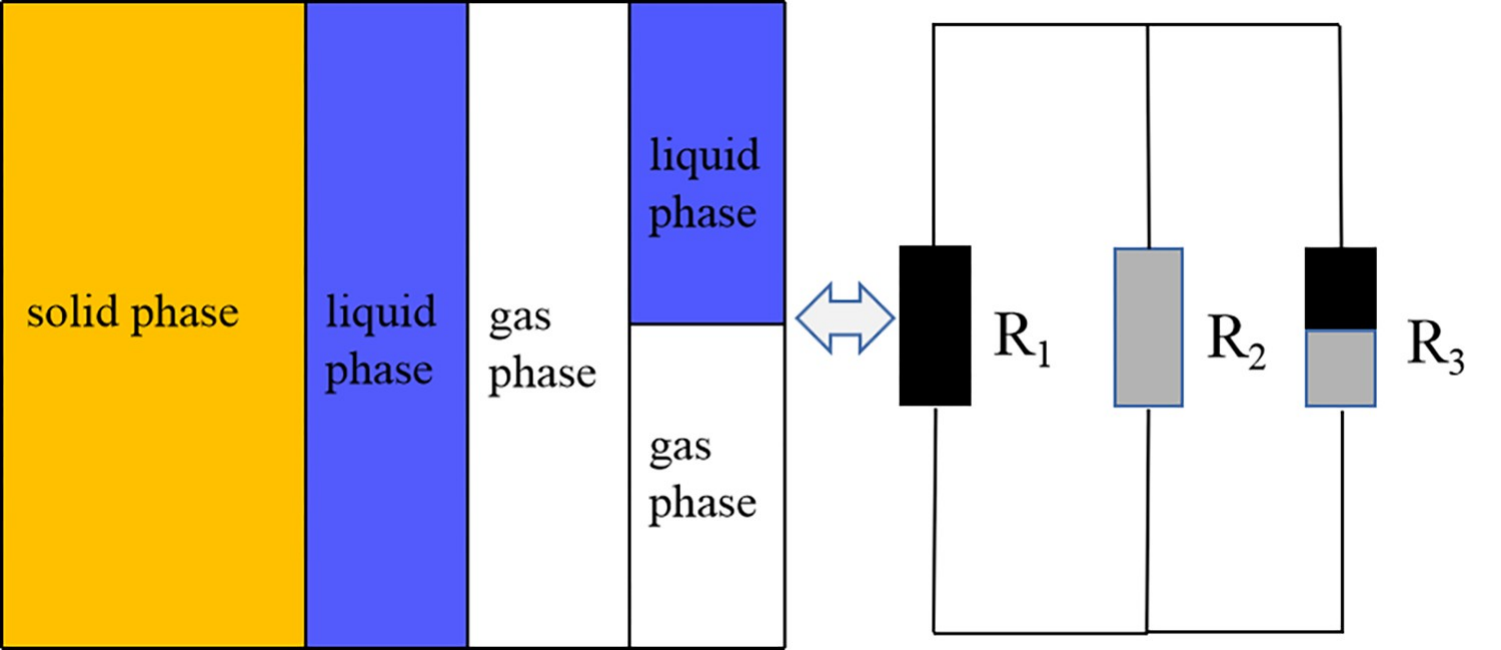
Diagram of triphasic conductive.

The rock-soil body of rainfall slope can be regarded as a three-phase combination of solid rock-soil, pore water and pore gas. When rainfall conditions and rock-soil body are determined, the water content depends on the porosity inside the slope. According to three-phase unsaturated rock soil resistivity model (Fig 11) [41]. The seepage process is essentially the displacement process of water and air during drying and wetting cycle. In the process of rainfall seepage, the slope gradually produces cracks, and the water accumulates to form a weak surface. The three-phase proportion of solid, liquid and gas in the slope changes, thus causing a significant change in resistivity.

Therefore, this paper taking the slope as a whole, according to Maxwell’s conductivity formula and three-phase unsaturated rock soil resistivity model, discuss the mechanism of resistivity change in each stage of rainfall slope [42].

According to Maxwell formula, as show in Eq (11):

$$\sigma_{0}=\sigma_{1}\frac{{3\sigma }_{2}-2\emptyset (\sigma_{2}-\sigma_{1})}{{3\sigma }_{1}+\emptyset (\sigma_{2}-\sigma_{1})}$$ (11)

Where, *σ*_1_, *σ*_2_, *σ*_0_ is the conductivity of medium 1, 2 and the overall medium respectively, ∅ is the proportion of medium 1 in the overall medium.

For dry rock-soil body before rainfall and saturated rock-soil body. According to *σ*_*A*_≪*σ*_*S*_、*σ*_*S*_≪*σ*_*W*_ and Eq (11), obtained Eqs 12 and 13:

$$\sigma_{R}=\sigma_{A}\frac{{3\sigma }_{S}-2\emptyset \sigma_{S}}{\emptyset \sigma_{S}}$$ (12)

$$\sigma_{R}=\sigma_{W}\frac{2\emptyset \sigma_{W}}{3\sigma_{W}-\emptyset \sigma_{W}}$$ (13)

Where, *σ*_*R*_ is slope conductivity, *σ*_*A*_ is the air conductivity in the pores and fractures, *σ*_*S*_ is the rock-soil conductivity of the ideal slope without cracks, *σ*_*W*_ is the conductivity of water, ∅ is the porosity.

For unsaturated rock-soil body, Maxwell’s formula continuously used for air, dry rock-soil body and air, water rock combination. According to *σ*_*A*_≪*σ*_*S*−*w*_, the relationship between conductivity *σ*_*R*−*w*_ and porosity ∅ of unsaturated slope can be obtained, as shown in Eq (14).


$$\sigma_{R-w}=\sigma_{A}\frac{{3\sigma }_{S}-2\emptyset \sigma_{S}}{\emptyset \sigma_{S}}\frac{{3\sigma }_{S-w}-2\emptyset^{\prime }\sigma_{S-w}}{\emptyset^{\prime }\sigma_{S-w}}$$ (14)

Where ∅′ is the proportion of residual air in the slope, σ_S−w_ is the conductivity of water bearing rock-soil, other symbols have the same meanings as above.

As ∅′<∅<1, the right side of Eq (14) tends to increase in the process of ∅ decreasing. The physical meaning is that during rainfall seepage, water displaces air, the proportion of air in pores decreases from ∅ in dry state to ∅′, and the conductivity increases.

According to the reciprocal relationship between resistivity and conductivity σ=1ρ, the relationship between slope resistivity and porosity in dry state, unsaturated state and saturated state can be obtained, as shown in Eqs (15–17).


$$\rho_{R}=\rho_{A}\frac{\emptyset }{3-2\emptyset }$$ (15)

$$\rho_{R-w}=\rho_{A}\frac{\emptyset }{3-2\emptyset }\frac{\emptyset^{\prime }}{3-2\emptyset^{\prime }}$$ (16)

$$\rho_{R}=\rho_{W}\frac{3-\emptyset }{2\emptyset }$$ (17)

Eqs (15–17) describe the whole process of the change of resistivity with seepage and fracture development during the slope from drying to saturation. It can be seen from Eq (15) to Eq (17) that the resistivity of the water bearing slope is positive correlation with the proportion of residual air, is negative correlation with the water content in the pores of the slope, which is consistent with the conclusion of Archie formula. The porosity increases with the development of cracks, decreases as water flows in. The conductivity of water is better than the conductivity of rock, and both of them are better than the conductivity of air. Air displaces water when cracks developing and the apparent resistivity increases when the proportion of air increases. Then the water displace the air and infiltrate along fractures in the progress of seepage, which cause the decrease of apparent resistivity. Therefore, the suddenly increase of apparent resistivity between measurement point 16 to point 18 after the 95th rainfall in this test can be interpreted as that the slope have damaged inside. After the 100th rainfall, cracks were observed between measurement point 16 to point 18 as shown in Fig 9, which can verify the rationality of the theory and test results. The phenomenon is consistent with the findings in reference[44]. The change of resistivity in the slope drying and wetting cycle is under the joint action of many factors (Chen et al 2020) [43]. This paper mainly studied the influence of drying and wetting cycle. Later research will further study the influence of other factors on the resistivity change.

### Damage location under drying and wetting cycle

Fig 13 show the damage location of slope under drying and wetting cycle. Waste dump slope has obvious heterogeneity and nonlinear characteristics, so it is often difficult to accurately predict the direction of seepage and fracture development. Taking the rainfall slope as a whole, when seepage and damage occur in the slope, the porosity, water content and saturation will change, and the corresponding resistivity will also change. It can be seen from the test results that the location of new cracks in the slope is close to the internal high resistivity anomaly area detected before rainfall. Under the influence of seepage water, fine particles in the high resistivity abnormal area are lost and slight local damage occurs. The stress unloading caused by damage concentrates the stress to the adjacent low resistance area, causing shear failure in the adjacent low resistance area and forming new tension cracks. The conclusion is consistent with the theoretical analysis in 4.3 and research result of [44].

**Fig 12:**
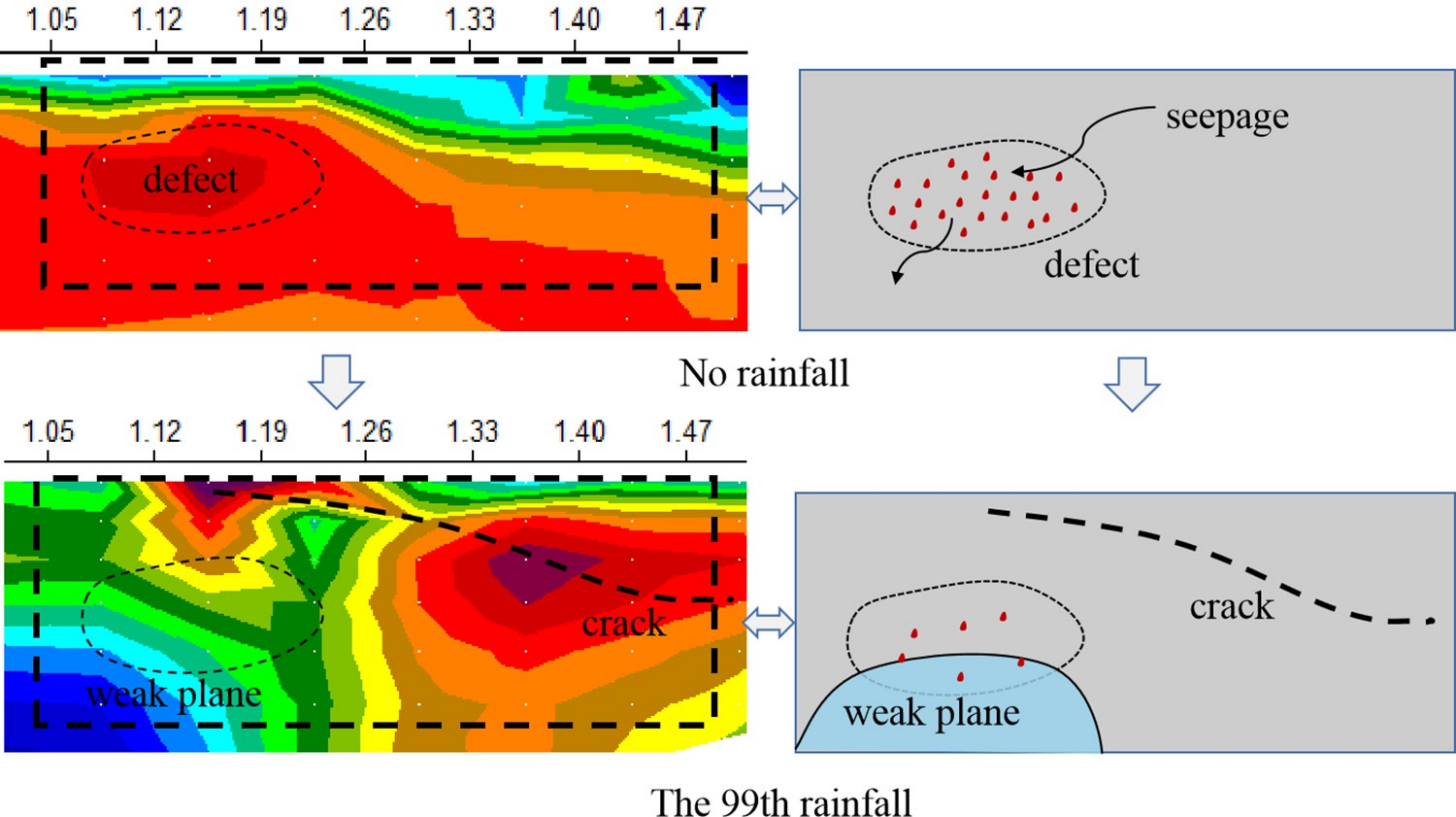
Location of rainfall slope damage.

Each layer of slope is almost saturated after the 95th rainfall. It can be judged according to (Fig 12) that the internal structure of the slope has changed irreversibly in the process of seepage erosion after saturation, and the slope body has been damaged to form fractures and saturated water bearing areas [45]. The resistivity shows regional mutation and fluctuation. The continuous saturated zone is formed by the connection of multiple saturated areas, the cohesion and friction angle of rock-soil body are reduced, and the slope strength is gradually reduced to failure. Therefore, the study considers that the high resistivity abnormal area in dry slope is potential damage area, and the apparent resistivity sudden change and abnormal fluctuation near the high resistivity abnormal area of saturated rock-soil body can be used as sign of slope damage, so as to predict the slope instability in advance.

### Inversion analysis of slope damage law

**Fig 13:**
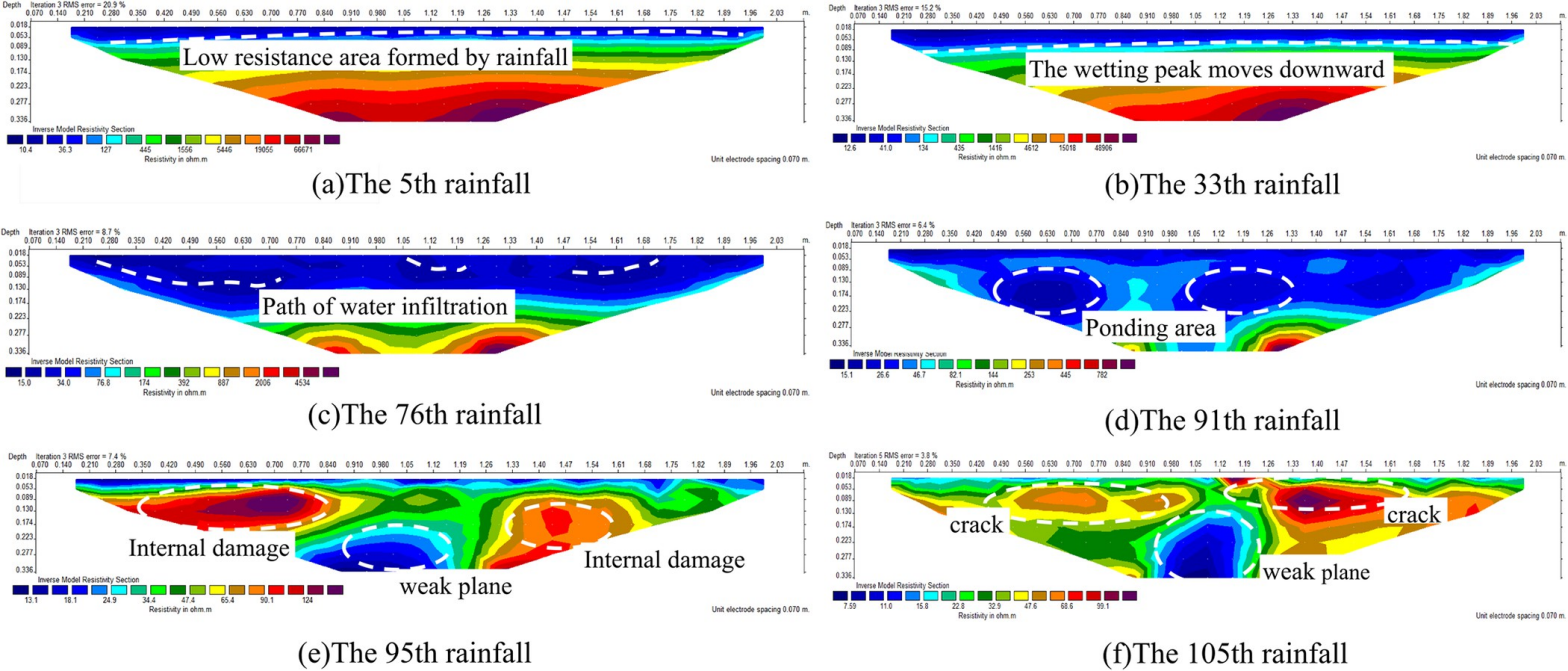
Seepage and crack development process at typical moment.

According to the change of apparent resistivity, selecte resistivity at typical rainfall time and draw inversion diagram to describe the law of seepage and fracture development in the survey line area under drying and wetting cycle, as shown in Fig 13.

In the initial stage of rainfall, there was no runoff on the surface, only rapid infiltration on the top of the slope. The resistivity distribution in the survey line area was clear without abnormal resistance. After the 5th rainfall, the cumulative rainfall was 10.5mm, resistivity of each layer in survey line area tended to be evenly distributed, the average depth of the wetting peak was about 5cm, and the lowest shallow resistivity was about 19 Ω·M. With rainfall process, water continuously seeped into the slope, ponding occured at the top of the slope, and shallow area of the slope reached saturation. No obvious cracks or damage on the slope top (Fig 13A).

After the 33th rainfall, the cumulative rainfall was 69.3mm. There was a significant difference in water infiltration on the slope. Resistivity of the shallow slope in the survey line area changed unevenly. The depth of the wetting peak on the left side of the slope was low, and the average depth of the wetting peak was 6cm. Water at the top of slope gradually diffused to the shoulder, infiltration rate at left side was faster than that in other areas. The results show that during the cycle of rainfall infiltration and measurement, left side of the slope produced cracks first, formed a water infiltration channel (Fig 13B).

After the 76th rainfall, the cumulative rainfall was 160mm, and the average depth of wetting peak was 21cm. Runoff erosion occured at many places on the slope surface, obvious tension cracks appeared at the rear of the slope top. Rainfall seeped into the slope through cracks at the top of slope. As shown in (Fig 13C), many obvious low resistance areas were formed inside the slope, indicated that rainfall seepage in different areas is uneven, water migration paths were formed inside the slope.

After the 91th rainfall, the cumulative rainfall was 191.1mm, and the moisture content of the soil mass in upper layer of slope gradually saturated. Under the influence of micro topography and other conditions on the slope, seepage in slope connected with each other, bearing capacity of water on soil particles was further enhanced, runoff were formed on the slope. Sliding occured at part of the slope. From (Fig 13D), the depth of wetting front moves down, and the infiltration rate of No. 14–16 measuring points in the central region was faster. There were many relatively high resistance areas and obvious low resistance areas in the slope. The high resistivity area was the crack formed by the expansion stress and shrinkage stress of the slope. The low resistance area was the water flow path and ponding area.

After the 95th rainfall, the cumulative rainfall was 199.5mm. There were many transverse cracks in the runoff area on the left slope, and many longitudinal cracks on the right slope. At the left toe of the slope, sliding occured due to rill erosion and increase of soil moisture content. As shown in (Fig 13E), a banded low resistance area inside the slope was connected with the water bearing area of the slope, a weak plane was gradually developed in the middle of the slope.

After the 105th rainfall, the cumulative rainfall reached 220.5mm. As the weight of the slope increased, Horizontal cracks on the top of the slope were further widened and gradually develop toward the shoulder of slope, network of cracks formed on the top of slope. With the increase of number and width of cracks, a large amount of water entered the slope. There were many crisscross cracks in the slope, traction sliding occured in the middle of the slope, the slope toe collapsed. As shown in (Fig 13F), within the range of No. 16–17 measuring points on the surface layer of the slope, obvious high resistance anomaly was detected, which corresponded to the position where cracks were found on the top of the slope. There were many high resistivity abnormal areas in the slope, indicated that with rainfall, seepage and evaporation cycle process, cracks were formed in the slope, and the slope was seriously damaged.

### Time-lapse inversion of slope damage law

**Fig 14:**
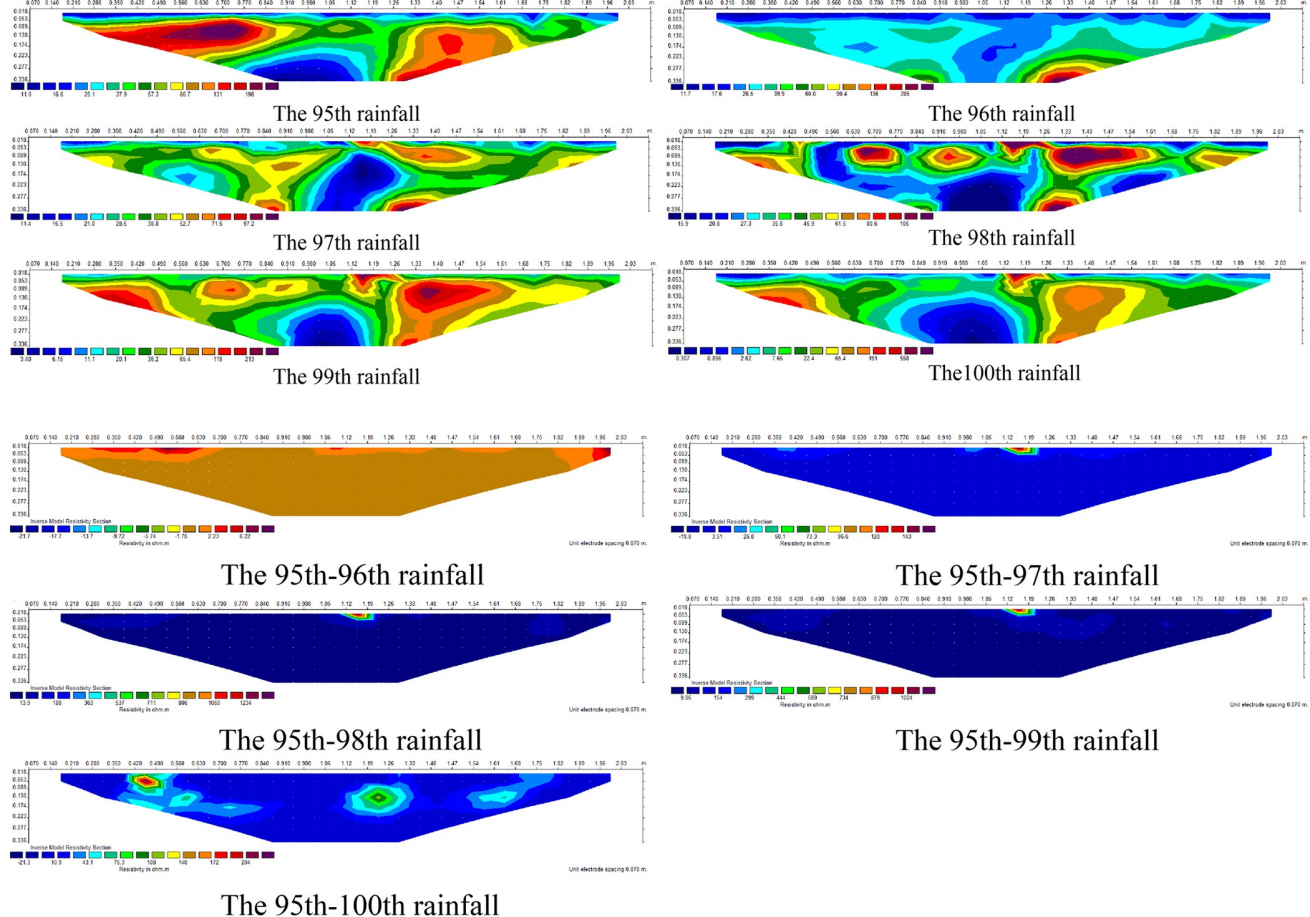
Inversion comparison of seepage and crack development.

In order to study the dynamic change of slope damage under drying and wetting cycle, according to change characteristics of apparent resistivity and development of cracks on the slope top, apparent resistivity of the 95th to 100th rainfall process is selected for time-lapse resistivity inversion, as shown in (Fig 14).

Obtain residual value *Δρ* according to Eq (8). The larger the residual value is, the greater the change of resistivity under the coupling of fracture development and rainfall seepage. This test took the apparent resistivity value after the 95th rainfall as the benchmark, and inverted the change of apparent resistivity value after the 96th to 100th rainfall. (Fig 14A) shows the apparent resistivity inversion after the 95th to 100th rainfall, and (Fig 14B)shows the time-lapse apparent resistivity inversion after the 95th to 100th rainfall. The variation range of residual value is -29Ω· M~118 Ω· M. The physical meaning is that there are cracks developed inside slope when the residual value is positive. The residual value is negative indicate that seepage or water accumulation were formed inside slope. It can be seen that there are two cracks developed within the abscissa range of 0.42m-0.49m and 1.05m-1.19m from (Fig 14B). The sensitivity of apparent resistivity change is better than cracks development on the top of slope, and the time-lapse resistivity can dynamically reflect the process of slope from damage to crack formation and later development.

In this study, both conventional resistivity and time-lapse resistivity analysis methods are used to carry out apparent resistivity detection at the same location at different times, analyze the dynamic change characteristics of the underground medium over time, reduce the multiplicity of inversion results, and apply to the underground medium with minor changes [46].

## Conclusion

A physical model of a waste dump slope under rainfall conditions was designed and established. The resistivity variation of the slope during rainfall was measured and the geoelectric field response characteristics of slope damage and disaster were discussed. The resistivity response formulas for slope seepage and crack development are derived, and the slope damage mechanism under drying and wetting cycle is studied. The internal damage law of the slope in the rainfall dump is obtained by inverse analysis. The research conclusions are as follows:

1.Based on the three-phase medium theory of rock-soil body and Maxwell conductivity formula, the expression of slope resistivity change during rainfall is derived in this study. There is a negative correlation between the resistivity and the water content of rainfall slope. The decrease of resistivity corresponds to the process of water replacement air and infiltration, and the increase of resistivity corresponds to the process of fracture development and enlargement of pore.

2.The sudden change point of resistivity of slope damage is found. The resistivity changes obviously after the 95th rainfall, and each layer of the slope near saturation. The abscissa position of the change corresponds to the fracture position formed on the slope surface. In the process of fracture development and formation, the resistivity of the soil in the nearby area changes in varying degrees, and the change time is earlier than the time when the fracture can be observed on the slope surface. The sudden change and fluctuation of resistivity after saturation can be used to characterize the water infiltration and fracture development during rainfall. In the next step of research, the mutation mechanism will be studied in combination with mechanics and physics.

3.Joint inversion of conventional resistivity and time-lapse resistivity can visually describe the subtle changes inside the slope, and reveals the dynamic evolution process of weak structural plane and fracture. The DC resistivity method and Electrical Resistivity Tomography can quickly and undamaged find the water migration and the development of small cracks in the slope. Provide basic information for multi-source prediction of rainfall slope instability. According to the change of resistivity, the advanced warning prediction of fracture development and slope failure can be realized under the condition of rainfall and seepage.

## Supporting information

S1 Data (ZIP)
